# On the mechanism by which dietary nitrate improves human skeletal muscle function

**DOI:** 10.3389/fphys.2015.00211

**Published:** 2015-07-29

**Authors:** Charles Affourtit, Stephen J. Bailey, Andrew M. Jones, Miranda J. Smallwood, Paul G. Winyard

**Affiliations:** ^1^School of Biomedical and Healthcare Sciences, Plymouth University Peninsula Schools of Medicine and Dentistry, Plymouth UniversityPlymouth, UK; ^2^Department of Sport and Health Sciences, College of Life and Environmental Sciences, University of ExeterExeter, UK; ^3^Institute of Biomedical and Clinical Sciences, University of Exeter Medical School, University of ExeterExeter, UK

**Keywords:** dietary nitrate, nitrite, nitric oxide, oxygen cost of human exercise, cellular bioenergetics, skeletal muscle mitochondria, coupling efficiency of oxidative phosphorylation, ATP turnover

## Abstract

Inorganic nitrate is present at high levels in beetroot and celery, and in green leafy vegetables such as spinach and lettuce. Though long believed inert, nitrate can be reduced to nitrite in the human mouth and, further, under hypoxia and/or low pH, to nitric oxide. Dietary nitrate has thus been associated favorably with nitric-oxide-regulated processes including blood flow and energy metabolism. Indeed, the therapeutic potential of dietary nitrate in cardiovascular disease and metabolic syndrome—both aging-related medical disorders—has attracted considerable recent research interest. We and others have shown that dietary nitrate supplementation lowers the oxygen cost of human exercise, as less respiratory activity appears to be required for a set rate of skeletal muscle work. This striking observation predicts that nitrate benefits the energy metabolism of human muscle, increasing the efficiency of either mitochondrial ATP synthesis and/or of cellular ATP-consuming processes. In this mini-review, we evaluate experimental support for the dietary nitrate effects on muscle bioenergetics and we critically discuss the likelihood of nitric oxide as the molecular mediator of such effects.

## Introduction

Inorganic nitrate (NO${}_{3}^{-}$) has long been considered an undesirable food component and pollutant of drinking water as nitrosation of secondary amines may produce carcinogenic *N*-nitrosamines (Magee and Barnes, 1956). However, the evidence that NO${}_{3}^{-}$ causes human cancers is weak and dietary NO${}_{3}^{-}$, as e.g. found in beetroot and spinach, has instead been linked to many physiological benefits (Gilchrist et al., 2010). Humans do not only get NO${}_{3}^{-}$ from their diet as it is also generated endogenously (Tannenbaum et al., 1978) by oxidation of nitric oxide (NO) formed canonically via the L-arginine/NO synthase pathway (Moncada and Higgs, 1993). Importantly, inorganic NO${}_{3}^{-}$ can be reduced to nitrite (NO${}_{2}^{-}$) and then NO, which offers an additional path of mammalian NO production that, unlike the canonical route, is independent of oxygen (O_2_) (Lundberg et al., 2008). NO is widely believed to mediate the benefits of NO${}_{3}^{-}$ (Lundberg et al., 2009) including protection against cardiovascular disease (Omar and Webb, 2014) and the metabolic syndrome (Carlström et al., 2010). It has recently been found that dietary NO${}_{3}^{-}$ lowers the O_2_ cost of human exercise as less respiratory activity is required for a set rate of skeletal muscle work (Larsen et al., 2007, 2010; Bailey et al., 2009, 2010). This finding is interesting as it challenges exercise physiology dogma that the steady-state O_2_ consumption for any individual is immutable at a given sub-maximal workload irrespective of age, fitness, diet, or pharmacological intervention (Poole and Richardson, 1997). This mini-review aims to evaluate the mechanistic understanding of NO${}_{3}^{-}$ effects on skeletal muscle function.

## O_2_ cost of human exercise

In a seminal publication, Larsen and colleagues reported that a 3-day supplementation with 0.1 mmol·kg^−1^ NaNO_3_· day^−1^ lowered pulmonary O_2_ uptake ($\dot{V}{\text{O}}_{2}$) by ~3–5% in humans completing sub-maximal cycling exercise (Larsen et al., 2007). Bailey and co-workers subsequently observed a 5% lower $\dot{V}{\text{O}}_{2}$ during low-intensity cycling exercise and a 16% improvement in the tolerable duration of high-intensity exercise over days 3–6 of a 6-day supplementation period with 5.6 mmol NO${}_{3}^{-}\cdot$ day^−1^, administered as 500 mL NO${}_{3}^{-}$-rich beetroot juice· day^−1^ (Bailey et al., 2009). Importantly, NO${}_{3}^{-}$-depleted beetroot juice does not improve exercise economy and performance (Lansley et al., 2011) eliminating antioxidants and polyphenols (Wootton-Beard and Ryan, 2011) as exclusive “active ingredients”. NO${}_{3}^{-}$-induced improvements have been observed in humans completing walking, running, cycling, rowing and kayaking exercise, and positive responses arise both acutely, i.e., 1–3 h after NO${}_{3}^{-}$ ingestion, and after prolonged NO${}_{3}^{-}$ supplementation over 3–15 days (Table 1). Acute (2.5 h post-ingestion) lowering of $\dot{V}{\text{O}}_{2}$ during low-intensity exercise is progressively greater at 4.2, 8.4, and 16.8 mmol NO${}_{3}^{-}$, whereas high-intensity exercise tolerance is unaffected by 4.2 mmol NO${}_{3}^{-}$, but acutely improved to a similar extent by 8.4 and 16.8 mmol (Wylie et al., 2013). Therefore, short-term supplementation (≥3 days) with at least 5 mmol NO${}_{3}^{-}\cdot$ day^−1^, or acute ingestion of at least 8.4 mmol NO${}_{3}^{-}$, might represent an effective dietary intervention to improve the economy and performance of human locomotion, at least in healthy, moderately fit adults (Porcelli et al., 2014). Since effects on resting $\dot{V}{\text{O}}_{2}$ are equivocal (Bailey et al., 2010; Kelly et al., 2013b; Larsen et al., 2014), NO${}_{3}^{-}$ benefits may be exclusive to contracting skeletal muscles.

**Table 1:** **Dietary nitrate improves the economy and/or performance of human locomotion**.

**Exercise**	**References^a^**	**Exposure period**	
		**1–3 h**	**3–15 d**
Cycling	Larsen et al., 2007—1st study reporting dietary NO${}_{3}^{-}$ benefit		✓
	Larsen et al., 2010	✓	
	Larsen et al., 2011		✓
	Bailey et al., 2009—1st study using beetroot juice as NO${}_{3}^{-}$ source		✓
	Vanhatalo et al., 2010	✓	✓
	Cermak et al., 2012		✓
	Wylie et al., 2013—Study reports dose-dependency of NO${}_{3}^{-}$	✓	
Running	Lansley et al., 2011—1st study using NO${}_{3}^{-}$-depleted beetroot juice as placebo		✓
	Porcelli et al., 2014		✓
Kayaking	Muggeridge et al., 2013	✓	
	Peeling et al., 2014	✓	
Walking	Lansley et al., 2011		✓
Rowing	Bond et al., 2012		✓

a *These studies are cited as examples—the list is not a comprehensive account of all available literature*.

Physiological NO${}_{3}^{-}$ effects appear muscle-fiber-type-specific as evidenced by improved perfusion (Ferguson et al., 2014) and calcium handling (Hernández et al., 2012) of murine fast-twitch type II but not slow-twitch type I muscle. Consistent with this, NO${}_{3}^{-}$ benefit on $\dot{V}{\text{O}}_{2}$ adjustment following the onset of exercise and on tolerance to high-intensity exercise is relatively large when the contribution of type II muscle fibers to force production is increased in human skeletal muscle (Breese et al., 2013; Bailey et al., 2015). These preferential physiological effects may relate to the comparably low microvascular P_O2_ in resting and contracting type II muscle (McDonough et al., 2005). Indeed, NO${}_{3}^{-}$ improves exercise economy and performance in hypoxia (Masschelein et al., 2012; Muggeridge et al., 2014) more markedly than in normoxia (Kelly et al., 2014). Importantly, NO${}_{3}^{-}$ attenuates the degree of exercise intolerance and the slowing of PCr recovery kinetics in hypoxia to the levels seen in normoxia (Vanhatalo et al., 2011). It thus appears that exercise economy and performance benefit most from NO${}_{3}^{-}$ when muscle O_2_ availability is low.

Although the majority of studies in healthy adults observe NO${}_{3}^{-}$-improved exercise economy and/or performance, the effects are attenuated in well-trained endurance athletes (Bescós et al., 2012; Peacock et al., 2012; Christensen et al., 2013; Boorsma et al., 2014; Hoon et al., 2014; Lane et al., 2014), inconsistent in diseased populations (Berry et al., 2014; Kerley et al., 2015; Leong et al., 2015; Shepherd et al., 2015; Zamani et al., 2015), and possibly different in aging humans (Kelly et al., 2013b). More generally, there is evidence of distinct NO${}_{3}^{-}$ responders and non-responders in many studies. The relative efficacy of dietary NO${}_{3}^{-}$ effects on skeletal muscle thus appears variable, which underscores the need for detailed mechanistic understanding. To aid such understanding, it is important to ascertain how the human body processes dietary NO${}_{3}^{-}$.

## Molecular fate of dietary NO${}_{3}^{-}$

**Figure 1:**
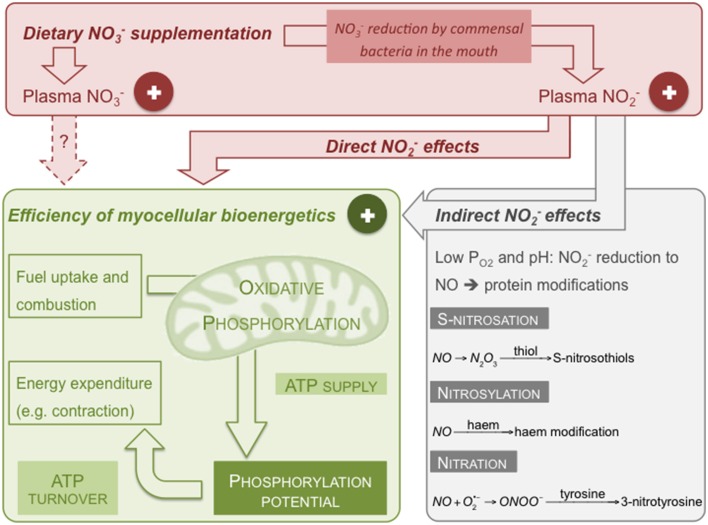
**Putative mechanism by which dietary NO${}_{3}^{-}$ may lower the O_2_ cost of human exercise**. Dietary NO${}_{3}^{-}$ increases plasma NO${}_{3}^{-}$ and NO${}_{2}^{-}$ levels thus improving efficiency of skeletal muscle ATP supply by oxidative phosphorylation and/or of ATP turnover. Effects on the bioenergetics of skeletal muscle cells may be direct or indirect through formation of NO. Shown reactions are examples of RNS-induced protein modifications (see text for details).

When humans eat NO${}_{3}^{-}$-rich food, NO${}_{3}^{-}$ is converted to NO${}_{2}^{-}$ by nitrate reductases in commensal bacteria that reside in the posterior part of the tongue (Duncan et al., 1995). Salivary NO${}_{2}^{-}$ is rapidly protonated in the acidic environment of the stomach resulting in the formation of NO and other reactive nitrogen species (RNS) including nitrogen dioxide (NO_2_), nitrous acid (HNO_2_), and dinitrogen trioxide (N_2_O_3_) (Benjamin et al., 1994; Lundberg et al., 1994; Lundberg and Weitzberg, 2013). NO${}_{3}^{-}$ ingestion increases plasma NO${}_{2}^{-}$ levels in human subjects (e.g., Lundberg and Govoni, 2004; Webb et al., 2008; Bailey et al., 2009; Vanhatalo et al., 2010; Kelly et al., 2013a). Possibly catalyzed by xanthine oxidase (Zhang et al., 1998; Li et al., 2003) and/or deoxyhaemoglobin (Cosby et al., 2003; Gladwin et al., 2004; Gladwin and Kim-Shapiro, 2008), NO${}_{2}^{-}$ is reduced to NO under conditions of low oxygen tension (Figure 1). Other sites of NO${}_{2}^{-}$ reductase activity include cytochrome *c* (Basu et al., 2008) and mitochondrial respiratory complexes III (Kozlov et al., 1999) and IV (Castello et al., 2006). However, mammalian NO${}_{2}^{-}$ reductase activity has only been shown *in vitro* and in animal models (Feelisch et al., 2008; Jansson et al., 2008), and under low P_O2_ (Li et al., 2001; Feelisch et al., 2008) and low pH (Modin et al., 2001). Generally, the low pKa of NO${}_{2}^{-}$ (3.34, Oxtoby and Nachtrieb, 1996) limits its physiological reduction, which is an inefficient process *per se* (Li et al., 2008) and thus requires high NO${}_{2}^{-}$ concentrations. Indeed, at physiological NO${}_{2}^{-}$ levels (see below), even *hypoxic* red blood cells do not liberate significant NO (Bryan et al., 2004). O_2_ competitively inhibits NO${}_{2}^{-}$ reduction by xanthine oxidase (Li et al., 2004) and oxygenated haem effectively scavenges free NO (Feelisch et al., 2008). NO may be converted to N_2_O_3_ that in turn may react with free thiols to generate S-nitrosothiols (Hess et al., 2005) via an S-nitrosation reaction (Figure 1). NO is also able to modify proteins through nitrosylation, e.g., via reaction with the haem of myoglobin (Ignarro, 1991). NO furthermore binds, in a reversible and O_2_-competitive manner, to the haem of cytochrome *c* oxidase, and in an O_2_-independent way, to the enzyme's active site copper (Giulivi et al., 2006; Brown and Borutaite, 2007; Cooper and Giulivi, 2007). Peroxynitrite (ONOO^−^) arising from the reaction of NO with the superoxide anion radical may undergo a nitration reaction with tyrosine residues to form 3-nitrotyrosine (Figure 1—Radi, 2004). Importantly, tyrosine-containing proteins are also nitrated in a myeloperoxidase-catalyzed reaction using NO${}_{2}^{-}$ and hydrogen peroxide (Marquez and Dunford, 1995).

Physiological NO${}_{2}^{-}$ levels range from 50 to 500 nM in human plasma (Bryan et al., 2004; Dejam et al., 2005; Feelisch et al., 2008) and a mean concentration of 12 μM has been measured in human skin (Mowbray et al., 2009). In rodents, NO${}_{2}^{-}$ concentration varies substantially between tissues, from below quantifiable limit in the liver, heart and lung to as high as 2 μM in kidney and 3.7 μM in lymph nodes; NO${}_{3}^{-}$ varies from 1 μM in the kidney to 50 μM in the aorta (Garcia-Saura et al., 2010). In humans, the ingestion of 10 mg NaNO_3_·kg^−1^ has been shown to increase mean plasma NO${}_{3}^{-}$ within 90 min from 30 to 432 μM and mean plasma NO${}_{2}^{-}$ from 123 to 392 nM (Lundberg and Govoni, 2004). Similarly, 500 mL beetroot juice containing 45 mM NO${}_{3}^{-}$ on average raises mean plasma NO${}_{3}^{-}$ to 380 μM and NO${}_{2}^{-}$ to 600 nM within 30 min and 3 h of ingestion, respectively (Webb et al., 2008). Plasma NO${}_{3}^{-}$ and NO${}_{2}^{-}$ reach peak concentrations, respectively, 1–2 and 2–3 h post-ingestion, and NO${}_{3}^{-}$ gradually returns to its base level after about 24 h (Ender et al., 1964; McKnight et al., 1997; Webb et al., 2008; Wylie et al., 2013). Mammalian tissue NO${}_{2}^{-}$ and NO${}_{3}^{-}$ both have *in vivo* half-lives of tens of minutes (Bryan et al., 2005). The half-life of NO${}_{2}^{-}$ in whole human blood is only about 110 sec (Kelm, 1999) as it is rapidly oxidized to NO${}_{3}^{-}$; the half-life of NO${}_{3}^{-}$ in blood is 5–8 h (Wagner et al., 1983; McKnight et al., 1997). About 60% of ingested NO${}_{3}^{-}$ is excreted by the kidneys (Green et al., 1981; Wagner et al., 1983).

The reliability of commonly reported NO${}_{3}^{-}$ and NO${}_{2}^{-}$ values very much depends on the assays used to measure these inorganic anions. The modified Griess reaction using sulfanilamide and N-1-napthylethylenediamine dihydrochloride is a frequently used assay for measuring NO${}_{2}^{-}$ (Tsikas, 2007). Plasma NO${}_{3}^{-}$ concentrations are readily determined using this spectrophotometric assay following NO${}_{3}^{-}$ reduction to NO${}_{2}^{-}$ by cadmium (Green et al., 1982) or vanadium salts (Miranda et al., 2001). However, the Griess test lacks the sensitivity to probe the nanomolar NO${}_{2}^{-}$ levels present in human plasma. Ozone-based chemiluminescence is a preferred method of detection, which often involves deproteinisation of plasma samples by zinc sulfate precipitation before analysis (Higuchi and Motomizu, 1999). NO${}_{2}^{-}$ measurement by chemiluminescence usually involves acetic acid/sodium iodide-mediated reduction to NO, which then reacts with ozone to produce a chemiluminescence signal (Bateman et al., 2002). NO${}_{3}^{-}$ can also be measured this way by reduction to NO via reflux of the sample in vanadium chloride at 95°C. Confounding the NO${}_{3}^{-}$/NO${}_{2}^{-}$ literature, in some assays NO${}_{3}^{-}$ is reduced to NO${}_{2}^{-}$ by bacterial nitrate reductases (Sun et al., 2003) whose activity varies from batch to batch. Confusing matters further, mere “NO_*x*_” values are reported to denote the sum of NO${}_{2}^{-}$ and NO${}_{3}^{-}$ levels. A last analytical note concerns the use of NO${}_{3}^{-}$-depleted beetroot juice as placebo control in *in vivo* studies (see Section O_2_ Cost of Human Exercise). It is important for experiments involving human participants to use a placebo juice that looks, tastes, and smells the same as the “real thing”. A placebo that meets these criteria can be prepared by passing beetroot juice through a Purolite a520e anion exchange column, which effectively and selectively removes NO${}_{3}^{-}$ (Gilchrist et al., 2013).

## Skeletal muscle bioenergetics

Dietary NO${}_{3}^{-}$ benefits on the O_2_ cost of exercise likely arise from increased efficiency of ATP synthesis and/or of skeletal muscle work (Figure 1). Indeed, NO${}_{3}^{-}$ increases the rate of human skeletal muscle PCr recovery after exercise in hypoxia suggesting an augmented maximum rate of oxidative ATP synthesis (Vanhatalo et al., 2011), and lowers the ATP cost of contractile force production (Bailey et al., 2010). These *in vivo* studies confirm that NO${}_{3}^{-}$ indeed affects skeletal muscle bioenergetics, but they do not disclose the underlying molecular mechanism. *In vitro* experiments with C2C12 myocytes show that beetroot juice provokes mitochondrial biogenesis and modestly increases basal cellular respiration without affecting respiratory capacity and proton leak (Vaughan et al., 2014). These observations indicate improved mitochondrial coupling efficiency as beetroot juice has increased the proportion of total O_2_ consumption coupled to ATP synthesis. Although the C2C12 experiments lack an appropriate NO${}_{3}^{-}$-depleted beetroot juice control (see above), increased coupling efficiency of oxidative phosphorylation agrees with data reported by Larsen et al. (2011), who show that skeletal muscle mitochondria isolated from NO${}_{3}^{-}$-supplemented subjects exhibit higher respiratory control and P/O ratios (defined in Brand and Nicholls, 2011) than mitochondria from non-supplemented controls, and that increases in P/O ratio correlate with NO${}_{3}^{-}$-induced decreases in whole-body O_2_ uptake during exercise. This increased efficiency of ATP synthesis in isolated mitochondria, however, emerges from decreased respiration linked to mitochondrial proton leak, not from stimulated O_2_ uptake coupled to phosphorylation (Larsen et al., 2011). NO${}_{3}^{-}$-lowered proton leak coincides with decreases in adenine nucleotide translocase protein and, to a lesser extent, uncoupling protein-3 (Larsen et al., 2011). It should be emphasized that these mitochondrial carriers do not necessarily contribute to proton leak (Nedergaard and Cannon, 2003; Vozza et al., 2014) and that leak is expected to account for little skeletal muscle respiration at low protonmotive force (Affourtit and Brand, 2006), i.e., the bioenergetic state attained during exercise. Dietary NO${}_{3}^{-}$ also lowers the apparent affinity of mitochondrial respiration for O_2_, an effect that is reproduced *in vitro*—acutely and pH-dependently—by NO${}_{2}^{-}$ (Larsen et al., 2011). Lowered affinity is attributed to an NO-induced rise in the apparent K_m_ of cytochrome *c* oxidase for O_2_ (Larsen et al., 2011) but, inconsistently, NO${}_{2}^{-}$ does not affect mitochondrial respiration or efficiency (Larsen et al., 2011) like NO is expected to (Brown and Borutaite, 2007). Apparent mitochondrial respiratory affinity for O_2_ depends strongly on the extent to which respiration is controlled by the enzyme reacting with O_2_ (Affourtit et al., 2001)—control of cytochrome *c* oxidase over O_2_ consumption may well have been affected by NO${}_{2}^{-}$ and pH, and also by dietary NO${}_{3}^{-}$-induced mitochondrial changes.

It remains to be demonstrated convincingly whether or not dietary NO${}_{3}^{-}$ effects in skeletal muscle are mediated by NO. Nitrite reductase activity requires high NO${}_{2}^{-}$ levels and exceptionally low P_O2_ and pH (see Section Molecular Fate of Dietary NO${}_{3}^{-}$) that may indeed manifest in the ischaemic heart (Brown and Borutaite, 2007; Hendgen-Cotta et al., 2010), but are unlikely in healthy muscle. In contracting muscle, myoglobin O_2_ saturation remains as high as 50% (Takakura et al., 2015) and although globins indeed exhibit nitrite reductase activity at this saturation (Huang et al., 2005), cytoplasmic NO will likely be scavenged by oxymyoglobin (Hendgen-Cotta et al., 2010). Even if O_2_ were sufficiently low for NO${}_{2}^{-}$ reduction in exercising muscle, we stress that dietary NO${}_{3}^{-}$ intake remodels skeletal muscle bioenergetics in the hours to days *before* exercise (see Section O_2_ Cost of Human Exercise), i.e., when the muscles are at rest. Importantly, NO${}_{2}^{-}$ also modulates cell signaling independently of NO in hypoxia and normoxia (Bryan et al., 2005). NO${}_{2}^{-}$ activates AMPK in rat aortic smooth muscle cells thus stimulating mitochondrial biogenesis, and increasing coupling efficiency and cellular respiratory control (Mo et al., 2012). NO${}_{2}^{-}$ activates PKA in cultured cardiomyocytes, stimulating mitochondrial fusion and again increasing cellular respiratory control (Pride et al., 2013). In both systems, NO${}_{2}^{-}$ improves efficiency of oxidative ATP synthesis without apparent effect on proton leak, which agrees with the beetroot juice effects on C2C12 respiration (Vaughan et al., 2014). NO${}_{2}^{-}$ also activates PKA in cultured adipocytes, increasing mitochondrial fusion, and stimulating glucose uptake (Khoo et al., 2014). Moreover, NO${}_{2}^{-}$ increases proliferation of muscle (Totzeck et al., 2014) and epithelial cells (Wang et al., 2012).

RNS can modify proteins (see Section Molecular fate of dietary NO${}_{3}^{-}$) and may thus improve mitochondrial coupling efficiency in various ways, e.g., by increasing proton translocation to electron transfer stoichiometries of respiratory complexes (*cf*. Clerc et al., 2007). By definition (Brand and Nicholls, 2011), coupling efficiency benefits from decreased proton leak and increased phosphorylation-linked respiration, as indeed reported by (Larsen et al., 2011) and (Vaughan et al., 2014), respectively. System-kinetic modeling furthermore suggests that substrate oxidation capacity, which is dependent on fuel and O_2_ availability, correlates positively with coupling efficiency (Affourtit and Brand, 2009). Dietary NO${}_{3}^{-}$ may thus improve efficiency of muscle ATP synthesis, at least in part, by increasing expression of glucose transporters (Jiang et al., 2014) and/or by raising insulin availability (Nyström et al., 2012).

Dietary NO${}_{3}^{-}$ increases the contractile force of fast-twitch mouse muscle by improving calcium handling (Hernández et al., 2012) suggesting the efficiency of ATP-demanding contraction may have increased. To our knowledge, no other data are available on the mechanism by which dietary NO${}_{3}^{-}$ affects ATP turnover. However, NO${}_{3}^{-}$ supplementation may also alter efficiency of other ATP-consumers and, importantly, the relative importance of dietary NO${}_{3}^{-}$ effects on skeletal muscle ATP supply and ATP turnover remains unclear. A systems-level functional analysis of cellular energy metabolism (*cf*. Brand, 1998) may shed light on these issues. Using myocytes isolated from human muscle biopsies (Nisr and Affourtit, 2014) the relative effects of RNS on ATP-generating and ATP-consuming fluxes—linked through the cell's phosphorylation potential (Figure 1)—may be identified and quantified in an unbiased manner. A challenge of such *in vitro* analysis will be the approximation of physiologically meaningful conditions, in particular the O_2_ tensions and energy demands that prevail during the development of dietary NO${}_{3}^{-}$ benefits.

## Conclusion

The striking benefit of dietary NO${}_{3}^{-}$ on the O_2_ cost of exercise is of obvious interest to athletes (Jones, 2014), but may also well impact on the quality of life of aging people suffering from muscle weakness and exercise intolerance. To rationally evaluate translational potential, our mechanistic understanding of dietary NO${}_{3}^{-}$ benefits on human skeletal muscle needs to be improved. By integrating biochemistry and physiology, and studying subjects at different age, it will be important to demonstrate which reactive nitrogen species mediate dietary NO${}_{3}^{-}$ effects at the cellular level, disclose all effects of nitrogen species on myocellular bioenergetics, confirm if they are direct or indirect via action on other tissues, and quantify the relative importance of these effects.

## Author contributions

CA wrote Sections Introduction, Skeletal Muscle Bioenergetics, and Conclusion, and produced the table and figure; SB and AJ wrote Section O_2_ Cost of Human Exercise; MS and PW wrote Section Molecular Fate of Dietary NO${}_{3}^{-}$. All authors edited and approved the entire manuscript.

### Conflict of interest statement

The development of a placebo beetroot juice preparation in PGW's laboratory was supported by James White Drinks Ltd. Otherwise, we confirm that this mini-review was written in the absence of any commercial or financial relationships that could be construed as a potential conflict of interest.

## Abbreviations

AMPK: AMP-activated kinase
PCr: phosphocreatine
PKA: protein kinase A
P_O2_: partial oxygen tension
RNS: reactive nitrogen species
$\dot{V}{\text{O}}_{2}$: pulmonary oxygen uptake.
